# Wearable Hardware Design for the Internet of Medical Things (IoMT)

**DOI:** 10.3390/s18113812

**Published:** 2018-11-07

**Authors:** Fayez Qureshi, Sridhar Krishnan

**Affiliations:** Department of Electrical, Computer, and Biomedical Engineering, Ryerson University, Toronto, ON M5B 2K3, Canada; krishnan@ryerson.ca

**Keywords:** wearables, hardware, internet of medical things (IoMT), electronic textiles, signal acquisition, edge computing

## Abstract

As the life expectancy of individuals increases with recent advancements in medicine and quality of living, it is important to monitor the health of patients and healthy individuals on a daily basis. This is not possible with the current health care system in North America, and thus there is a need for wireless devices that can be used from home. These devices are called biomedical wearables, and they have become popular in the last decade. There are several reasons for that, but the main ones are: expensive health care, longer wait times, and an increase in public awareness about improving quality of life. With this, it is vital for anyone working on wearables to have an overall understanding of how they function, how they were designed, their significance, and what factors were considered when the hardware was designed. Therefore, this study attempts to investigate the hardware components that are required to design wearable devices that are used in the emerging context of the Internet of Medical Things (IoMT). This means that they can be used, to an extent, for disease monitoring through biosignal capture. In particular, this review study covers the basic components that are required for the front-end of any biomedical wearable, and the limitations that these wearable devices have. Furthermore, there is a discussion of the opportunities that they create, and the direction that the wearable industry is heading in.

## 1. Introduction

Technology advancements in recent years have caused an explosion of smart and highly connected computing devices that are in a small form factor. Nearly all of us have encountered some sort of communication device, whether it be in entertainment, health, or lifestyle [1]. The miniaturization of hardware played a significant role in this advancement; however, a lesser-known factor was the Internet [1,2]. The internet played one of the most important roles in this technology advancement, and it is mainly due to the transformation it has gone through since the 1990s. During that time, the Internet was used primarily for communication purposes (emails); however, all that changed in the 2000s with the introduction of wireless technologies, such as the mobile phone [2]. Billions of users were now connected to the Internet, and this eventually lead to the ongoing phenomenon called the Internet of Things (IoT). The IoT connects items, such as robots, sensors, and actuators, to the internet [2]. When these items are worn by a person they are called wearables, and usually allow the individual to manage their health and safety. Getting into the proper definitions, the IoT is simply defined as a network of physical objects that are supported by sensors and embedded technology for data communication that allow for interaction with the environment [3]. Furthermore, wearable devices are defined as devices that can be worn by an individual and can continuously monitor an individual’s activity without interruption [3,4]. These wearables use sensors, such as tri-axial accelerometers, magnetometers, altimeters, and gyroscopes, to create an intuitive virtual environment [3,5].

**Figure 1:**
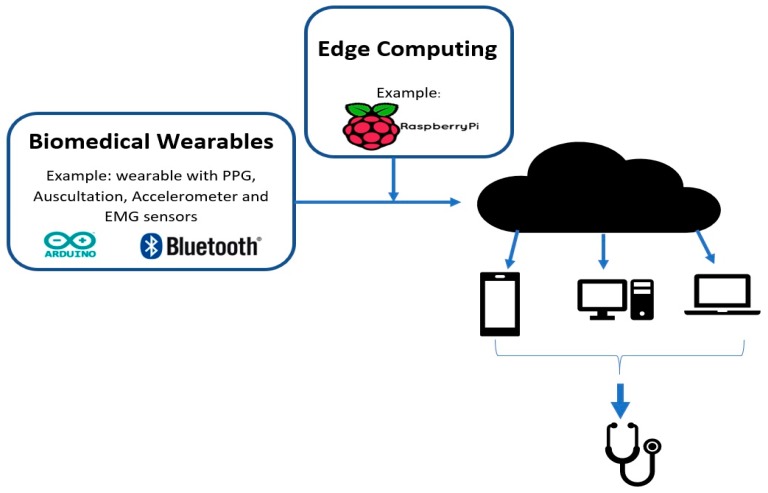
This figure shows the Internet of Medical Things (IoMT) architecture. Logo sources: [6,7]. PPG, photoplethysmogram; EMG, electromyogram.

Due to the fast-paced ongoing development that is occurring in the IoT domain, it is important to understand some of the key terminologies and their variations that are used by engineers and the Industry. We start with the Internet of Wearable things (IoWT); simply put, this is the combined use of the IoT and wearable devices. As development continues on wearables, they start to incorporate advanced features and we start to transition into the Internet of Wearable Things [2]. It is evident that wearables significantly benefit from the IoT, and, hence, we see that they have already made it into many aspects of our lives, such as in fashion, health, and entertainment [1,2]. Some well-known examples of IoWT devices in the market are computerized watches, such as the Samsung Gear and the Apple watch. They can gather information, such as a user’s step count, pulse rate, kilometers travelled, and calories burnt [5]. One distinction to consider is that these devices are lifestyle devices; however, the Health Industry is one of the most promising industries for IoT applications. In the same way as the IoWT, when wearables are used in medical applications, it creates another term and an industry called the Internet of Medical Things (IoMT). Biomedical wearables are then the devices used in the IoMT and, thus, function through the cloud to perform complex tasks. An architectural diagram is shown in Figure 1 below. This industry is pushing towards minimizing the size of the wearable while capturing more vital signs, and finally sending reliable and secure data [3].

Biomedical wearables are especially important when considering the fast-paced lifestyle of most working-class people nowadays. A significant portion of the day is spent commuting between tasks, and often health and fitness are overlooked. In addition to that, the time-consuming task of going to the doctor for a diagnosis, a prescription, and then the treatment results in many people avoiding the doctor or going to a clinic until it is absolutely necessary [3]. This is why a significant amount of research is being done on ways to monitor a patient’s health and send the physician real-time data of their health parameters [3]. As a matter of fact, the implementation of these wearables creates a preventative approach to medicine where individuals can monitor their own biosignals longitudinally. This can also be referred to as telehealth, and, with the aid of the IoT, telehealth has become a prominent market. Telehealth, or telemedicine, is simply defined as the integration of communication technologies and healthcare, where technology is used as a medium to deliver the healthcare services [8]. Physicians or health staff can then remotely monitor the patient’s condition and provide medical suggestions and aid. Finally, data can also be stored on the cloud or secure servers for later use.

To understand the significance of the IoT, wearables, and telemedicine, we will be looking at some crucial facts and numbers. The IoT is said to be the next trillion-dollar industry, and The Global Wearable Technology market has grown significantly since 2012. In 2012, it was 750 million and now, in 2018, it is worth 5.8 billion [4]. Furthermore, U.K.-based research projects estimated a 10-fold increase in the number of wearable devices being shipped in the last 5 years. They found that the number increased from 13 million in 2013 to 130 million in 2018 [4]. In North America alone, the wearable device market is said to reach out to 385 million users [5]. Currently, in the market, there are 429 wearable devices and they have an average price of $326 USD. Within this, there are only 87 medical devices, since the majority of wearable devices are targeted towards lifestyle and fitness [9]. Moving on to The Global Telehealth and Telemedicine Market, it was worth 34 billion in 2015, and is projected to increase to 79 billion by 2020 [10,11]. This is why, in the last decade, wearable devices, the IoT, and telemedicine have attracted major attention from the academic community, governments, as well as the industry, which has led to their current popularity. For example, in 2009, GE and Intel Corporation worked together to create the Intel Health Guide. It is a home-based telehealth technology that focusses on aiding seniors that live independently and patients with chronic care to manage their care and symptoms from their homes [11]. As can be seen, there is a significant interest that will continue to develop for research units in providing new and innovative products as well as improving the currently available products.

With that in mind, our review study is focused on investigating the possible biomedical wearable designs for non-invasive applications. Specifically, we are investigating the front-end hardware components that are required for the design of these wearables and a short discussion on how they can be used for analyzing clinical conditions. Some typical bio-signals that can be obtained from these wearables include: photoplethysmogram (PPG), electromyogram (EMG), electroencephalogram (EEG), movement data (such as gait) and body sounds. This review study is organized as follows: Section 2 will provide a brief background on the signals, important distinctions, and factors to consider before designing a wearable. Section 3 will introduce the necessary components for designing working biomedical wearables. We will investigate their specifications as well as look at a possible modality that can be used for data transfer. Section 4 will discuss some of the limitations that these wearables have as well as some of the advantages. Section 5 will conclude this review with a review on opportunities for textile wearables, and the direction the wearables industry is heading in.

## 2. Background and Factors to Consider

### 2.1. Sample Biomedical Signals

The body generates many waveforms, and they can be captured from various parts of the body using different sensors. The four commonly used signals for biomedical wearable purposes are outlined in this section. Table 1 includes their frequency ranges.

#### 2.1.1. Photoplethysmogram (PPG)

Photoplethysmography (PPG) technology has been at the center of the recent development of biomedical wearables, whether it be in medical or lifestyle applications. This is primarily due to the need to measure heart rate variability effectively. Traditionally, to obtain accurate Heart Rate Variability (HRV) data, an electrocardiogram (ECG) signal is used, and HRV is measured as the variations in the peak-to-peak time interval for successive cardiac cycles. This is also known as the R-R interval. In diagnostics, HRV analysis provides significant information on the sympathetic and parasympathetic function of the Autonomic Nervous System (ANS) [14,15]. Hence, it is important to measure HRV; however, there are some limitations to acquiring the ECG signal. Firstly, it requires at least three electrodes positioned at specific anatomical positions. Secondly, ECG instruments are not suitable for daily use at home and also require trained technicians and nurses for use. Finally, the electrodes may cause irritation to the patient’s skin [12]. This is why significant effort has been made to measure HRV by using PPG. Moving on, another important parameter than can be captured by PPG is the atrial blood oxygen saturation level of the patient, also known as SpO2 [16]. Oxygen saturation (SpO2) is the percent of oxygen-saturated hemoglobin when compared to the rest of the blood. Blood in the body is either oxygenated or deoxygenated, and they both have different light absorption characteristics [12,16]. This is an important physiological parameter for monitoring blood circulation and respiration.

**Figure 2:**
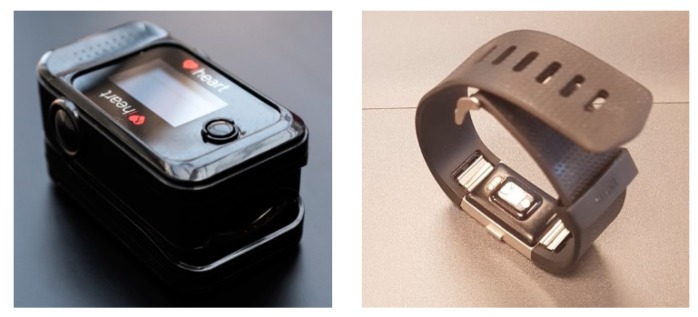
This figure shows the finger-tip (iHeart) sensor on the left and the wrist (Fitbit Charge 2) sensor on the right Image source: [18].

Having understood the significance and importance of PPG, the simple definition of photoplethysmogram or PPG is that it is a non-invasive optical technique to measure blood volume changes in the microvascular bed of tissue [14]. The basics of how an PPG sensor works is a light-emitting diode (LED) light penetrates the skin, light travels through the tissue, and then the signal is received by the photo detector. The most common locations to detect PPG signals are the finger-tip and at the wrist. Depending on which location is chosen, different techniques need to be used to detect the signal from the finger-tip or wrist. There are two types of techniques: Transmittance and Reflectance. Transmittance detects the light transmitted through the tissues by a photodiode that is kept opposite of the light source, and is commonly used in fingertip-type sensors. Reflectance detects the intensity of reflected light using a photodiode that is kept on the same side as the light source, and is commonly used in wrist band applications [17]. Some common examples of this are the iHeart device that clamps on to your finger and the Fitbit device that detects the signal from the wrist shown in Figure 2 below. [18,19]. Moving on to light sources, there are three different types: Green, red, and infrared. Green LEDs have been shown to be more accurate for sensing heartrates, while red and infrared LED light sources are mainly used to calculate oxygen concentrations because of their different penetration intensities [15]. The reflections that are measured by the PPG sensor are highly associated with the variations in the blood perfusion of the tissue, and are used for obtaining heart-related information of the cardiovascular system [14]. Another advantage to using PPG is that it can be applied to any blood-bearing tissue, and it is entirely non-invasive. The importance of this signal is in fact due to the low cost, user flexibility, and the portability of the sensors that are used to capture the PPG [14]. Estimating heart rate from PPG is challenging due to motion artifacts in PPG signals. These motion artifacts can be caused by respiration, blood pressure oscillation, circadian biorhythm, and thermoregulation. Some popular techniques used to measure HRV are: performing a power spectral analysis of the peak-to-peak interval and the finite harmonic sum model using an accelerometer to capture motion artifacts [15,17]. 

#### 2.1.2. Electromyography (EMG)

For the past few decades, surface EMG, or just EMG, has been successfully used in medical and research applications that allow for the diagnosis of a wide range of motor and neural conditions [20]. EMG is a technique that measures the response of a muscle when an electrical stimulation is applied by the nerves [5]. This electrical stimulation is known as the action potential (AP), and the signal (EMG) at the skin’s surface is the summation of the electrical activity of the motor unit action potential (MUAPS). There are two ways to measure EMG: one is by an invasive approach that uses a needle, and the other is a non-invasive approach that uses dry or wet electrodes on the surface of the skin [5]. Acquisition of these surface EMG signals is usually done by surface sensors that lead to the inputs of a differential amplifier. In clinical applications, an EMG is captured using dedicated medical instruments that are composed of silver-chloride (Ag/Cl) electrodes in combination with a conductive gel. The gel reduces the contact impedance with the skin, and this system is able to capture extremely high-quality signals that allow for the diagnosis of muscular and neural human systems [20].

**Figure 3:**
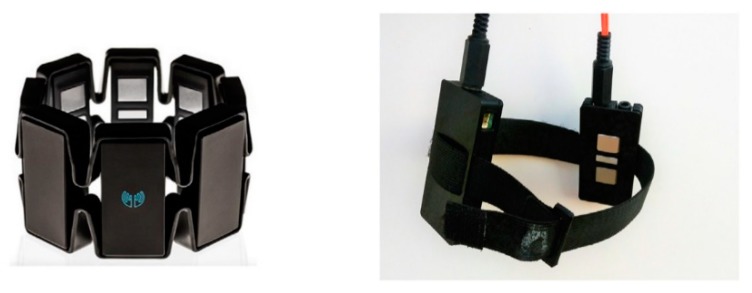
This figure shows the Myo band on the left and two EMG channel sensors on the right. Image source: [21,22].

On the other hand, wearables cannot use Ag/Cl electrodes as they are limited in terms of their form factor and power consumption. Accordingly, they usually employ dry electrodes. Besides that, dry electrodes also have an advantage over the Ag/Cl electrodes in long-term use while still providing comparable signals to the Ag/Cl wet electrodes [5,20]. A few factors that affect the ability to detect surface EMG signals are: skin perspiration, the distance between the active muscle fiber and the sensing site, crosstalk between adjacent muscle fibers, and signal variability that is caused by sensor impedance [20]. These factors need to be considered in clinical applications as well as wearables. On the other hand, an important factor to consider when detecting EMG signals is that it varies greatly with sensor placement, and locating the ideal place to position the sensor is necessary [5]. To add to this, an EMG signal that is acquired from the same muscle but with an electrode that is placed at a different location will result in different results that affect the analysis [5]. This is because a standard mapping protocol has not yet been developed for EMG sensors in addition to the fact that the ideal location changes from patient to patient, so it has to be adjusted for each patient individually [5,21]. In common EMG wearable applications, active sensors are used for signal capture. These sensors use three electrodes: two metal electrodes are used for differential acquisition of the signal, and one is used as a reference electrode [20]. Then, the signal is passed through an on-board miniaturized circuit with amplification and signal conditioning. It is then integrated and passed to a micro-controller unit (MCU) for analog-to-digital (A/D) conversion. These sensors can be used for applications that range from clinical diagnostics all the way to prosthetic control [20]. Looking now at some wearable designs using EMG, we see examples, such as the Myo band, that use eight EMG channels. The Myo band detects the signal by placing the sensor securely above the muscles that generate the signals in the forearm, allowing it to detect five gestures (finger spread, fist, wave in, wave out, and double tap) [21]. However, a recent study, done in 2018, proves that only two EMG channels—one placed on the wrist flexor and other placed on the wrist extensor—are sufficient to classify four of the same five hand gestures as the Myo band [21]. What this demonstrates is that the EMG sensors need to be placed right above the muscle being investigated. That is, it is subjective; however, a way to determine the correct location is by verifying the signal beforehand using an oscilloscope. This is a normal process done by rehabilitation experts in the industry [21]. Figure 3 shows the Myo band and the 2 channel EMG sensor below. 

#### 2.1.3. Auscultation of Body Sounds

The first act that a medical professional performs in a diagnosis is auscultation, and that alone demonstrates its importance. Auscultation refers to the act of listening to the human body, and is usually done by using a Littmann stethoscope [8]. Therefore, cardiac auscultation refers to listening and interpreting heart sounds. It is a very cost-effective approach as well as being non-invasive and easy to use. Moreover, it is the most used technique by doctors for the primary screening of early cardiac illnesses [8]. Cardiovascular diseases are one of the major causes of death in the world; hence, it is important to evaluate cardiac functions using a fast, non-invasive technique [23]. The main heart sounds include S1 and S2. S1 occurs at the onset of ventricular contraction and corresponds to a QRS complex in an ECG. Electrocardiogram (ECG) represents the electrical activity of the heart and the QRS complex are the most visual peak and troughs of that ECG signal. The complex consists of 3 deflections labeled Q, R and S respectively. The second heart sound, S2, corresponds to the closure of semilunar valves and finally S3 and S4 can sometimes be heard as well as other clicks and snaps. Normal heart sounds range from 20 to 420 Hz [8]. Auscultation serves as the first point of diagnosis and vital sign monitoring. This is because listening to these sounds can reveal information regarding the status of the underlying functions. For example, heart sounds often reveal abnormalities if a murmur is present and allow for the detection of disorders of the body. Furthermore, there is a strong connection between the heart and the lungs, when compared to the rest of the body’s systems, which is known as the cardiopulmonary system. Therefore, when analyzing the cardiac system, it is common to consequentially analyze the lungs and breathing of the patient. This is referred to as lung auscultation, which is used to detect respiratory disorders. Lung sounds can be classified into three categories: normal, abnormal, and adventitious. Normal sounds are quiet and hardly audible; abnormal sounds refer to the lack of these normal sounds; and adventitious sounds refer to wheezing and crackles that are strong indicators of disease [8].

**Figure 4:**
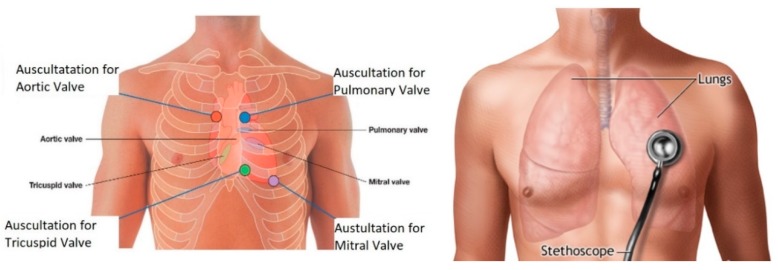
This figure shows heart sound auscultations and lung sound auscultation locations. Image source: [26,27].

Moving on, understanding how to detect these sounds is another important consideration. It is vital to understand not only the anatomy of the heart and lungs themselves, but also the surface anatomy. Surface anatomy refers to understanding the landmarks on the surface of the body that can be used to locate organs inside the body. Using surface anatomy, one can palpate the ribs and start to determine the auscultation regions for the heart [24]. The best sound will not necessarily appear right above the heart; rather, the ideal location for cardiac auscultation is based on the direction of the blood flow and the orientation of the valves [24]. With this understanding, the aortic valve is best heard at the 2nd right intercostal space, the tricuspid vales is best heard at the left lower sternal boarder in the 5th intercostal space, the pulmonary valve is heard best at the 2nd left intercostal space, and, finally, the mitral valve is best heard in the 5th intercostal space at the midclavicular line seen in the figure below [24]. Finally, normal lung sounds are heard in the chest and are primarily limited by the distance between the primary sound generation site and the stethoscope [25]. The detection region varies, and can include from the collar bone to the bottom of the rib cage as shown in Figure 4.

#### 2.1.4. Gait Analysis

The importance of a gait analysis is evident in the fact that, each year, there are around 3.2 million deaths worldwide because of physical inactivity. As a matter of fact, physical inactivity leads to chronic disease and disability [28]. On the other hand, regular physical activity is associated with health improvements in many populations. Therefore, the importance of measuring and encouraging individuals to be active is evident. Moreover, a clinical mobility assessment fails to mimic the real world requirements; for example, the 10 m walk test underestimates gait velocities [28]. As a result of this, it is important to quantitatively assess mobility in real-world environments. Step counting is the most common measure of physical activity. Sensors that provide this need to be highly accurate, lightweight, and allow for use in homes and the community. The crucial part is to mimic real-world daily activities, as it is unrealistic and an oversimplification to assume that individuals walk consistently at high speeds. One disadvantage of the current step detection algorithms and sensors is the decreased accuracy at slower speeds, and these slower walking speeds are the main indicators of movement disorders [28].

**Figure 5:**
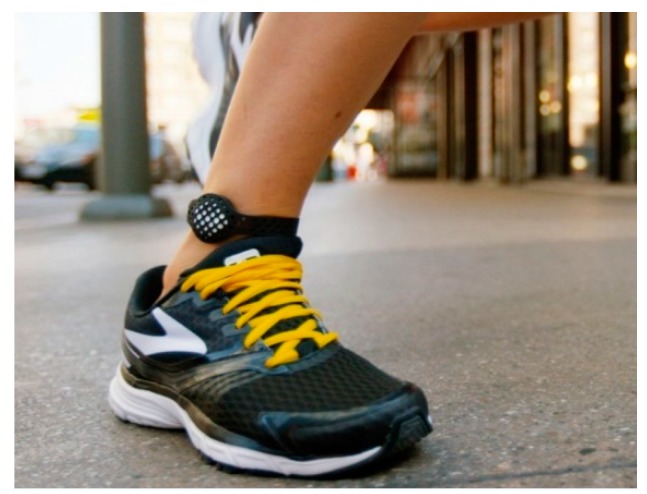
This figure shows the Moov Now device that contains nine-axis motion sensing. Image source: [29].

Detecting the physical activity of humans is possible with the placement of sensors, usually accelerometers and gyroscopes, on different parts of the body. There are different approaches to this, and the most common ones that are seen nowadays are pedometers, which are watches or sensors that are placed on the wrist for activity tracking. However, one limiting factor for these devices is accuracy. Therefore, sensor location needs to be updated, and a study done in 2014 proved that step counts can accurately be obtained from three-axis accelerometers placed on the thigh, waist, and ankles [28]. These systems performed well in low-gait-speed scenarios and outperform commercial pedometers. With this, there are several examples of activity trackers in the market and an example is shown in Figure 5.

### 2.2. Important Distinction: Medical versus Non-Medical Wearables

One of the most important distinctions that will determine the hardware and software requirements of biomedical wearables is the difference between medical and non-medical biomedical wearables. The term “medical devices” is loosely defined in Canada by the Food and Drugs Act as “a wide range of health or medical instruments used in the treatment, mitigation, diagnosis, or prevention of a disease or abnormal physical condition” [9]. Some common examples are artificial heart valves, pacemakers, synthetic skin, and medical laboratory diagnostic instruments. These devices are evaluated and monitored in Canada by the Therapeutic Products Directorate (TPD) and in the United States by the Food and Drug Administration (FDA). The TPD is a national authority that determines the effectiveness and quality of diagnostic and therapeutic medical devices in Canada. There are four classes that any device for sale is classified and grouped into. The first class is the lowest potential risk class (Class 1), and an example of a device that belongs to this class is a thermometer. The last class (Class 4) is highest potential risk class, and an example of a device that belongs to this class is a pacemaker. Class 1 products do not require a license, while the rest of the classes do, and the review process becomes more onerous as you move into higher classes [9]. Two items can be issued: either a Medical Device or an Establishment License. The process is similar to that of the FDA in the U.S., and many of the rules are adopted by the FDA. Further information can be found in the Medical Devices Regulations of the Food and Drugs Act of Canada [9]. On the other hand, fitness, lifestyle, or non-medical wearables are those that do not intend to provide any diagnosis, mitigation, or treatment of a disease. This is further supported by the FDA, who states that the concept for determining if a device is medical or non-medical is based on the intended use [30]. The technologies of both medical and non-medical devices can be the same, so both devices can measure the exact same signals with the same quality; however, if a device is analyzing the data to provide treatment, it will be considered a medical device and will have to go through an extensive process for approval [30]. To put it in another way, non-medical devices are not useful in diagnosing most medical conditions but can be used to track fitness and allow for patient-centered disease prevention.

### 2.3. Factors to Consider

The design of any biomedical wearable starts with a firm understanding of the biosignal being measured. For example, for an ECG signal, it can be easy for experts to identify the QRS due to its distinguishable characteristics that can be used in an analysis. However, that is not the case for all biomedical signals, and a considerable number of transformations is required to extract information from the signal [5].

#### 2.3.1. Four Design Factors

When the design of a wearable commences, whether it is for medical purposes or non-medical purposes, there are some factors to consider. Those factors are: (1) the characteristics of the measured signal; (2) the human factors; (3) the economic costs, and, finally; (4) the environment that the device will be in [5].

Starting with the first factor, it requires an understanding of the signal generation source and the properties of the signal. For biomedical applications, signals that are captured by sensors are commonly retrieved from the skin and are responses to the electrical stimulation of the nerves and muscles. Alternatively, the properties of the signal include several aspects, such as: determining whether the signal being recorded is reliable, whether there are usable signal-processing techniques for the signal, whether the signal is stationary to allow for analysis, and whether the signal needs compressive sensing algorithms at the acquisition stage [5]. This is important, because, based on these factors, a decision on which sensor to select will be made during the design phase to allow for signal capture without any information loss. The second factor, medical risks, requires an understanding of the user of the device and the interactions they have with the device. This means considering the environment the device will be used in, what materials the device should be made of, safety requirements, and how the device will affect the patient’s daily life [5]. For example, in a hot environment, the patient will be subject to more sweating than usual: will that change the obtained signal? Moreover, does the material the wearable is composed of cause any allergic reactions, pain, or discomfort? Finally, do the patients deem the device to be obstructive or overly complicated, and prefer not to use it? These factors are important, since a convenient device that is both hardware and software friendly will be used more often by the patient than one that is not. This is also referred to as the technology adoption rate, and is a considerable downfall for many devices. The third factor, economic costs, refers to designing an instrument that is affordable, is compatible with existing technologies, and is readily available. The designed device should be affordable and should not require extensive changes to their existing technologies. Furthermore, it should be available to purchase on many platforms, so patients do not have to go to specific locations to buy them. The importance of this factor arises because lifestyle or non-medical wearables can be relatively cheaper than medical wearables, since they do not go through an extensive process for approval. The last factor, the environment, refers to the environmental noise the device will face when in the real world. For example, the signal-to-noise ratio is a crucial measure of how effectively the device can capture the signal and reduce the noise.

Considering and meeting these factors does not guarantee a successful product, and they are only for lifestyle or nonmedical wearable devices and not medical devices. The FDA in the United States and the TPD in Canada regulate all medical devices extensively, and these devices must go through a long process that comes at a significant cost and requires time to ensure that the devices are safe for consumers.

#### 2.3.2. On-Chip and Edge Computing

While the majority of IoT devices in the market use some sort of Cloud Computing, it is not the only option. Rather than using the current popular way for transferring data to a separate personal computer (PC) to be sent to the cloud for data analysis, lightweight tasks can be performed on the micro-control unit itself. This is limited by the processor on the board, how much memory is available, and if the micro-control unit has storage capabilities. Some Arduinos contain enough memory to perform lightweight tasks and computations on the chip.

**Figure 6:**
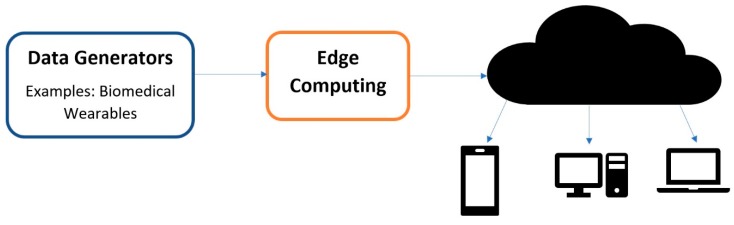
This figure shows the Edge Computing architecture.

An extension of this is called Edge Computing, which performs computations at the edge of the internet [31]. As the shift towards IoT and IoMT continues, large quantities of data of exceptional quality will be generated by all of the devices surrounding us. It is estimated that around 50 billion things will be connected to the internet by 2020, and by 2019 people and machines will be producing 500 zettabytes of data while the global data center’s (Internet Protocol) IP traffic will only reach 10.4 zettabytes [31]. This means that all of these things and devices will be producing data at a much higher rate than can be processed by the cloud. As a matter of fact, growth in the bandwidth of networks has come to a standstill, and the speed of data transportation has become the main bottleneck for cloud-based computing [31]. This means that most of the data produced by IoT and IoMT devices will never reach the cloud. Therefore, the simple definition of edge computing is any computation that occurs between the data-generating devices and the cloud data centers [31]. This includes processes such as data offloading, data storage, data processing, and IoT management, as shown in Figure 6. This also improves user privacy, since the data that are sent for edge computation are normally private when compared to the cloud.

## 3. Biomedical Wearables and Their Components

### 3.1. Allocation of Hardware Design

**Figure 7:**
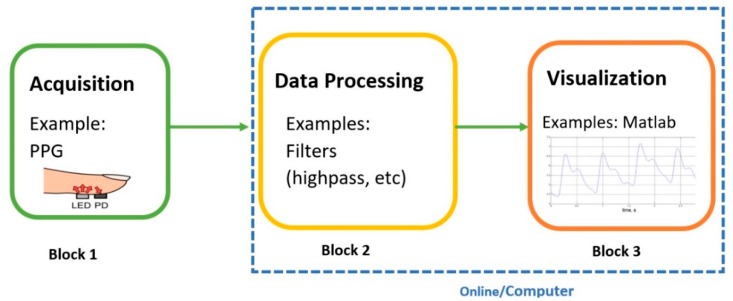
This figure shows the three phases to any wearable. LED, light-emitting diode; ADC, analog-to-digital, PD, photo detector. PPG image source: [32].

The design of any electrical device in the industry usually utilizes a team-oriented process. This means that teams are assigned a certain task that they must complete in order to create a final product. Hence, just like any other electrical device, the design and prototyping of a biomedical wearable starts by a similar team-oriented process. This creates effective teams that are focused on a common goal and use characteristics such as positive team interdependence, group accountability, and teamwork skills. In academia, analogous approaches are employed on a smaller scale where each block in the figure below represents a team and a task. Normally, a couple of students are assigned to each block (and consequently a task). The breakdown of each project varies and is very diverse; however, for biomedical and electrical engineering purposes generally, there are three important blocks or teams to be considered: Signal Acquisition, Data Processing, and Visualization of the data. The teams work together on a common goal while each individual team is focused on their own goals. This area shown in Figure 7 and the first block, Block 1, is the Acquisition phase, where raw data are captured from the human body. This is done by the sensor that is placed on the integument (skin) of the body. Block 2 is the data processing block, where digital filters and further data conditioning, such as segmentation, de-trending, and feature extraction can be applied. Block 3 is the visualization of the data using a user-friendly technique. Blocks 2 and 3 are commonly performed on a personal computer with the aid of software (MATLAB) in basic prototyping applications. In IoT applications, Blocks 2 and 3 can be done on the cloud or on a server, and this is subject to change depending on the application.

**Figure 8:**
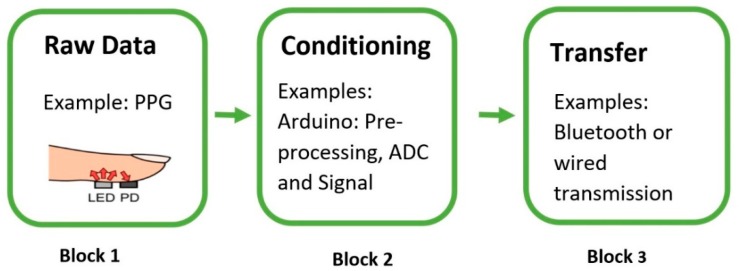
This figure shows the three blocks of the Acquisition Phase. PPG image source: [32].

The following figure, Figure 8, focuses on the element to be covered in detail here: Acquisition. As mentioned above, acquisition refers to the front-end of the project and includes all of the hardware and software that is required for signal capture. Figure 8 below demonstrates the three main components that are necessary to acquire raw data from the human body. Block 1 of Figure 8 deals with the raw data that are captured from the sensor. Since most signals are analog, there needs to be an analog-to-digital (ADC) conversion and a way to communicate with the sensor to allow for data transfer, and that is Block 2. For this, it is vital to have open-source and low-cost tools and platforms. The most common platforms for this are Arduino and Raspberry Pi, and they have played a transformational role at the outset of biomedical wearables. The last part, Block 3, is sending the data to a server to be sent to the cloud or for analysis. Commonly, Bluetooth modules are used or Arduinos with inbuilt Bluetooth or Wi-Fi capabilities. The next section will focus entirely on the hardware used to construct the Acquisition Phase. 

### 3.2. Hardware Requirements and Methods

Based on the current market in 2018, there are four sensors that stand out from the rest and those will be the focus of this review. Supporting those sensors for conditioning and transfer will be a micro-control unit (MCU, e.g., an Arduino Uno), a HC-05 Bluetooth module, and a Raspberry Pi Zero W. The four sensors include: an electret microphone, a PPG Pulse Sensor, an analog EMG sensor, and the MPU9250. The specifications for each sensor can be found in Table 2. It is important to note the motivation behind choosing these sensors. Firstly, they are commercially available at a low cost, and have significant support that ranges from online troubleshooting tutorials to full demo codes. As a matter of fact, the chosen sensors are specifically popular for prototyping. This means that they are specifically designed to reduce complex front-end circuitry by integrating them into the sensor. This makes most of these sensors “plug-and-play”-type sensors that allow for easy prototyping and development at any skill level. As a bonus point, they have software support for Arduino specifically so that full demo codes are available that can be modified easily using the available datasheets for different projects. Secondly, any one of these sensors can be individually used to monitor a certain medical condition or can be combined to allow for monitoring and diagnosing a variety of medical conditions. An example is using an EMG sensor to detect muscle diseases or using it with the inertial measuring unit (IMU) as a two-step validation system.

Moving on, once the raw data has been captured by the sensors, it can then easily be manipulated for analysis in IoT applications using the Raspberry Pi Zero W as a webserver or an edge computing device. A battery source is required for powering the wearable, and Arduino boards allow for several possibilities. However, the power source used for these wearables will be a 9 V Alkaline battery that is readily available in every supermarket. It will provide the Arduino with enough voltage and current to run the Bluetooth module and the sensors. The following subsection goes over the details and the advantages that each sensor possesses over its competitor sensors.

#### 3.2.1. PPG Sensor Description and Bioinstrumentation

Starting off with the Pulse Sensor, it is used to capture PPG signals and these signals can be captured from the fingertip or the wrist. This sensor is made by World Famous Electronics IIc, and is called the ‘Pulse Sensor’. It uses a bright green LED, and is a plug-and-play sensor that captures, amplifies, and cancels noise by circuitry on the sensor. It does not have inbuilt ADC; therefore, it is important to convert this analog input signal to digital using the Arduino Uno. Further, the operating voltage allows for easy interoperability with other components and can function from 3.3 to 5 V using only 4 mA at 5 V [37]. Finally, their website (pulsesensor.com) contains several “Getting Started” projects with the full Arduino code. One example of a project is the blinking of an LED with a heartbeat. The project also provides access to the raw data by the Serial Plotter on Arduino IDE.

**Figure 9:**
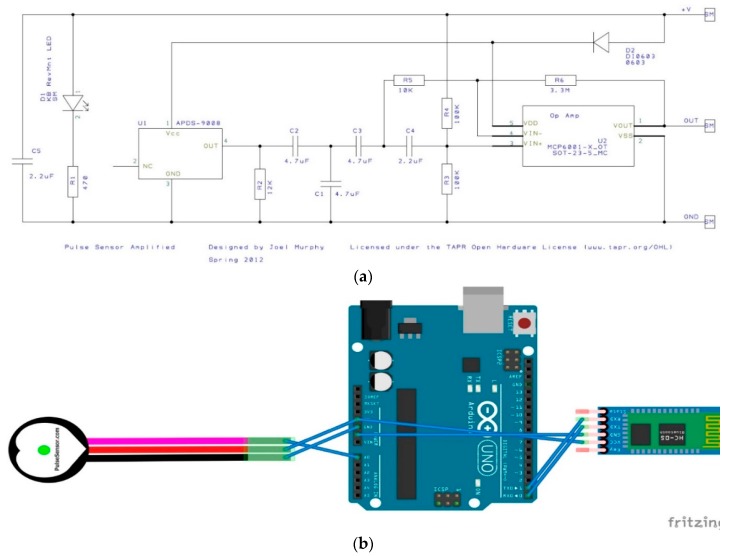
(**a**) This figure shows the internal circuitry of the pulse sensor. Image Source: [29]. (**b**) This figure shows the front-end connections of the pulse sensor.

As mentioned above, the sensor is designed to be a “plug-and-play”-type sensor. What this means is that only three wires are needed to connect the sensor to the Arduino, and no external circuitry is required to operate the sensor and retrieve the PPG signals. Furthermore, the sensor itself is an open-source hardware project, and, hence, a schematic of the internals is available and is shown in Figure 9a. To connect the sensor, the three wired connections are: ground, power, and the analog signal, and these are visible in Figure 9b as well as in Table 3(a). The sensor itself was secured to the index finger using the supplied Velcro straps. Moving on, the software side of the front end required us to take advantage of the online support available on the Pulsesensor website [29]. First, the Arduino Library was downloaded from the Arduino IDE. Next, a provided example in this library, the GettingStarted Project, was used to blink the Arduino’s built in LED with a baud rate of 9600. Finally, to visualize the signal, the Serial Plotter on the Arduino IDE was used, and further changes can be made to this code based on the application.

#### 3.2.2. EMG Sensor Requirements and Description

**Figure 10:**
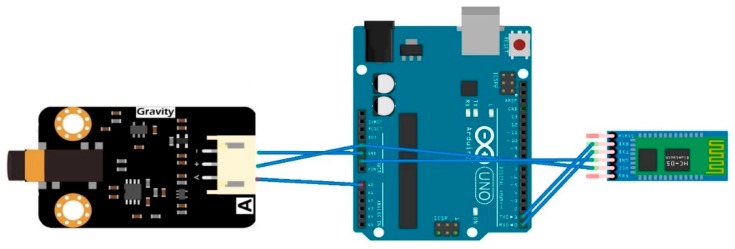
This figure shows the front-end connections of the EMG sensor.

Following that, the EMG Sensor is produced by OYMoiton and it also does not contain inbuilt ADC. The sensor captures the surface electromyograph (sEMG) signals that reflect muscle and neural activity in humans [35]. This too is designed to be a “Plug-and-Play” sensor, and, thus, it includes on-board circuitry that amplifies the signal within the range of ±1.5 mV and attenuates noise, such as power interference. The sensor was secured to the forearm flexor muscles by using the supplied straps. The current design will use only one EMG channel; however, two EMG channels can be used. Similarly, this sensor requires three connections to function properly with the Arduino, and they are the ground, power, and analog signals shown in Figure 10 and Table 3(a). It is important to note that there are two separate boards that comprise this sensor. Figure 10 shows the second board, which is called the Signal Transmitter Board, and from this board the supplied probe wiring connector is used to connect to the dry electrode, which is then secured to the forearm flexor muscles. Furthermore, just like the pulse sensor, OYMoiton’s website contains tutorials and sample code for the Arduino IDE. Taking advantage of this, the EMGFilters library was first installed, and then the code was run at a 115,200 baud rate. The sEMG signal is then visible on the Serial Plotter, and further changes can be made to this code based on the application.

#### 3.2.3. Microphone Requirement and Description

**Figure 11:**
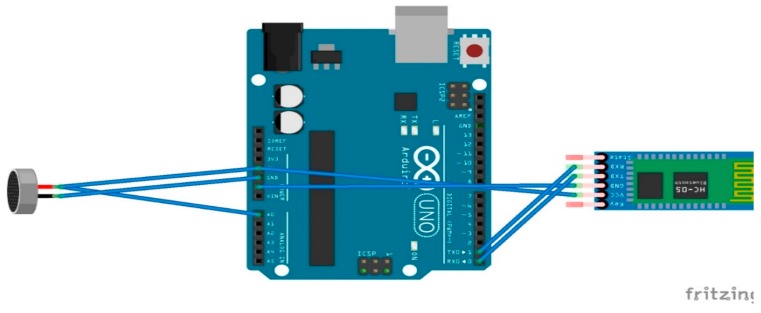
This figure shows the front-end connections for the microphone.

Next is the electret microphone, which is used for capturing heart and lung auscultation based on the locations mentioned in the Background section. The chosen electret microphone has a frequency range from 100 to 10,000 Hz [33]. It is produced by Challenge Electronics, and has a minimum sensitivity-to-noise ratio of 58 dB. This device results in an analog input and, hence, it also requires the use of an MCU, such as the Arduino Uno board. The microphone is connected directly to the Arduino’s analog pins, and the ADC is done on the Arduino. No further circuitry is used in the detection of the signal. As for the sensor placement for proper signal capture, the anatomical positions that are used by medical professionals for conventional stethoscopes were used as mentioned in the Background section. Finally, a simple code for retrieving an analog signal from the Uno was used at a 115,200 baud rate. Figure 11 below shows the hardware connections that are required for this sensor, and Table 3(b) demonstrates the front-end connections.

#### 3.2.4. IMU Requirements and Description

**Figure 12:**
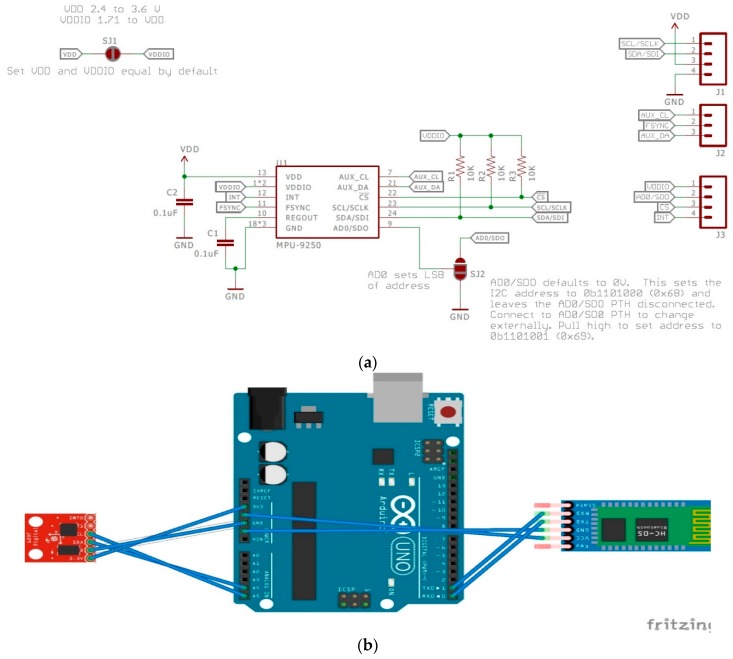
(**a**) This figure shows the schematic for the inertial measuring unit (IMU). Image Source: [38]. (**b**) This figure shows the front-end connections for the IMU.

Last is the inertial measuring unit (IMU), which is called the MPU9250. This device is manufactured by IvenSense, and is a multichip module that consists of a three-axis gyroscope, a three-axis accelerometer, and a three-axis magnetometer [34]. Communication with all of its registers can be done using I2C or SPI, and it has its own Digital Motion Processor. The Digital Motion Processor (DMP) allows for motion processing and low-power gesture recognition through the purchase of IvenSense Apps to program the device. Unlike the pulse sensor and EMG sensor, there are no tutorial codes or “getting started” projects offered by the manufacturer. However, it is a widely used sensor, and Sparkfun offers a great “Hookup Guide” that includes libraries and example code [38]. Taking advantage of that, first the library was installed using the Arduino IDE for the MPU9250. Next, the MPU9250BasicAHRS_I2C code was used and run at a 38,400 baud rate, and further changes can be made to this code based on the application. As for the placement of the sensor, the IMU will be placed on the ankle for motion detection using the nine-axis motion detection capabilities it offers. Figure 12a below shows the internal components of the IMU, and Figure 12b shows the hardware connections that are required for this sensor. Finally, Table 3(b) shows the hardware connections that are required for proper function.

#### 3.2.5. Arduino and Raspberry Pi Description

In a sense, the most important device is the Arduino, as it constitutes the connection between the sensor and the IoT aspects of this review. The Arduino will be used to communicate with the sensors and the Hc-05 module, and will supply power to these sensors and boards. It will require coding, and much of it is available through the online support that was mentioned above for the individual sensors and for using the Bluetooth module [6]. The only other important aspect to consider for the Arduino is that it can function by various power sources, such as a 9 V battery with a snap connector, an A-B universal serial bus (USB), or an alternating current (AC) to direct current (DC) adapter. Finally, the IoT aspect of the design will require the use of a Raspberry Pi Zero W. This can be used not only for sending data to the cloud, but also for edge computing as mentioned in the Background section. This is one of the cheaper computers that the Raspberry Pi Foundation produces. An additional module, called ADC Pi, can be bought and added to the Raspberry Pi for additional conversion if needed, and it comes with Bluetooth Low Energy, Bluetooth 4.1, and 802.11 b/g/n wireless LAN. Further, it comes with a 1 GHz single core CPU, 512 MB RAM, a CSI (Camera Serial Interface) camera connector, and micro USB and mini HDMI (High-definition Multimedia Interface) ports [7].

## 4. Analysis/Discussion

### 4.1. Hardware Analysis

Current healthcare systems still rely on visits to the doctor, monitoring that uses cumbersome equipment, and doctors’ experience-based prognosis [5]. Using the “Connected Human” idea, this review aims to demonstrate the possibility of developing low-cost and clinically relevant hardware designs to be used for integrated healthcare. Moreover, these devices have the potential to be used as a medical wearable for the tele-monitoring of patients that are either in hospitals, clinics, or assisted living. The goal is not to replace the doctor or their disease prognosis, but to share the data by cloud services between doctors, patients, and relevant staff. With these goals in mind, we have managed to design wearables that can accomplish that to an extent. These devices are currently not medically approved by the FDA or regulated. However, these devices have the potential to capture clinically comparable signals that can be analyzed, stored, and transferred over the cloud. Moreover, there is great potential to use these designs as stepping stones into designing medical wearables and re-designing these devices into commercially ready products. One interesting observation is the importance of understanding and following the Factors to Consider section. What is meant by this, for example, is that the Pulse Sensor provides a plug-and-play experience that is capable of executing the complex task of calculating heart rate variability from PPG signals. This is an added benefit of this product over other comparable sensors, and it uses the Ponicare Plot of the Inter-Beat Interval values to calculate HRV. Furthermore, these medical wearables have complete Arduino code in their repository, which allows for adjustments and changes to be made to the code by the user. Hence, the availability, support, and documentation for this sensor is immense, which could only have been found with research beforehand while utilizing the “Factors to Consider” section. Moving on, the electret microphone does not contain any circuitry for signal amplification and conditioning. Therefore, the manual use of op-amps, resistors, and capacitors will allow for adaptive filters to be used.

Focusing now on the IoT and telemedicine portion, the choice of sensors was based on simplifying the acquisition phase with the least number of steps. Hence, sensors that have on-board signal conditioning, are available for purchase, and that are easy to use were preferred. Furthermore, in IoT applications, the Raspberry PI plays an important role. It can be connected to all or some of these sensors through Bluetooth. This wireless connectivity by Bluetooth to the sensors and by Wi-Fi to the cloud provides the flexibility of connecting anywhere there is a wireless connection. The cloud, as mentioned above, has become a vital part of telemedicine and the IoT. Data are usually stored either in disparate clouds or in siloed systems that limit data analysis [4,31]. Firstly, some of these IoMT devices have the potential to produce large quantities of data; for example, capturing an 8-h stream of EEG and EMG signals for sleep monitoring. This cannot efficiently be done, since these files will be several gigabytes in size each night, and that could be a heavy load for networks. Secondly, some of these IoMT applications require very short response times, since it might be a life and death situation, which is again limited on the cloud. Finally, these sensors will measure data that might be private and not suitable to be sent to the cloud, and, hence, cloud computing might not be an efficient choice for these applications. Consequently, a tool such as Raspberry Pi is necessary for these applications. Such a tool can be used to transport the data to the cloud or for performing edge computing before the data are sent to the cloud, which will significantly reduce the number of computations that will need to be done on the cloud. It is a small, inexpensive, yet powerful computer board that can perform many tasks that desktop personal computers are capable of. They have been used in many telemedicine applications where a compatible webcam module that is capable of 1080p videos is used to stream microscopic images to remote locations [39].

A relevant example of these biomedical devices currently being used successfully in a country is Finland. They, much like many other countries, have a passionate start-up community that is dedicated to personalized healthcare solutions [40]. However, where they differ is that their official health technologies range from X-ray and imaging equipment to wearable technology that can analyze an individual’s heartbeat or alter an athlete’s training [41]. This led to the creation of a national patient data repository that covers the public and private healthcare sectors. They were indeed one of the first countries in the world to create this, and it allows every Finnish person to access their health records and prescription history [41]. This demonstrates the potential of wearables, and their probable future in the medical domain once they are approved for use in other countries.

### 4.2. Discussion

The success of these wearables, whether it be for medical purposes or non-medical purposes, relies heavily on human factors. As discussed in the Factors to Consider section, these factors need to be considered; however, even if all of these factors are met, the wearables are still restrained by independent problems that can arise. The creation of highly connected devices that can transmit information between themselves and to the online world leads to crucial questions and problems. The two most prominent problems that arise are: technological issues and security issues [4,11]. These points can single-handedly determine the fate of a wearable, and this demonstrates their importance. Hence, the Institute of Electrical and Electronics Engineers (IEEE) has been working, and continues to work, towards several antidotes to these problems.

Starting off with technological issues, these mainly deal with the interoperability between devices and whether the data that the devices obtain are accurate and dependable. Since all of the sensors that were mentioned above are manufactured by different manufacturers, they do not have a default standard interface and protocol. This is evident in our project, as it leads to the use of an Arduino with a Bluetooth module for each sensor individually, and not the integrated Digital Motion Processor in the MPU9250. Usually, different sensors from different manufacturers have different characteristics, and, therefore, the captured signals vary. The IEEE has some projects underway for this concern, the first being a Conformity Assessment Program and the second is the 2510 Standard Project [42]. The first project works with independent test labs and manufacturers to determine if the device will meet IEEE standards, while the second is more focused on the sensors that are used in Internet of Things applications and works on creating quality metrics for data that are retrieved from sensors that are used in the IoT.

In terms of security issues, wireless security breaches will definitely be an ongoing concern, specifically with regard to patient privacy and how it will be protected. Starting with some basic protection that everyone has access to, Bluetooth technology does support Advanced Encryption Standard-128 built-in encryption. There is active improvement currently underway on this technology, as more wearable devices are entering the market that require up-to-date security [4]. Furthermore, since Raspberry Pi is a Linux system, most of the common security measures that are available for Linux are available. However, further improvements need to be considered to limit attacks and the potential loss of data that the Raspberry Pi collects. Some known and tested security issues with Raspberry Pi are: (1) the Raspbian operating system has a secure shell protocol on port 22. This protocol is used when there is a need for a remote log on, and if it is left on it could lead to breaches; (2) The Raspbian operating system has a default user that allows access to the device. This should be deleted rather than ignored or a new user made as anyone who knows this could access the device [43]. Moving on to some higher-level security, in 2017 the IEEE Standards Association announced an initiative that is designed to protect digital identity for the global online community [42]. It is called “Industry Connections Programs: Digital Inclusion through Trust and Agency”, and will support their other programs, such as the IEEE Blockchain Special Interest group and the IEEE Internet Initiative on internet governance. The Industry Connection Program aims to build a consensus for standards, codes of conduct, and certifications to protect consumer and patient data [42]. It will be a universal digital identification system that does not rely on any legal documents and will, therefore, protect the user’s personal data. Efforts such as these by the IEEE are fundamental to the success of medical wearables and non-medical wearables. Without proper regulatory approvals, devices will not be trusted by individuals and that can lead to their downfall.

Perhaps the most interesting aspect brought up by this review is the wide array of diseases that can be diagnosed with the combinational use of these sensors. For example, PPG signals contain information relating to heart rate, SpO2, blood pressure, and respiratory rate, and sensors can measure these with medical grade quality as mentioned in the Background section. Therefore, the PPG signal can be used to diagnose anxiety, atrial fibrillation, hypertension, sleep apnea, pregnancy, stress, and stroke [44]. Moving on to activity tracking, it allows for the diagnosis of diseases such as Arthritis, Bipolar disorder, Parkinson’s, Restless Leg Syndrome, Seizures, and Insomnia [44]. Following that, auscultation can allow for diagnosis of diseases such as the common cold and heat stroke that causes changes in breathing. Lastly, EMG can be used in combination with auscultation to diagnose diseases relating to the cardiac system and neuro-muscular disorders [5]. If these four sensors are combined into one wearable, it will allow for the diagnosis of up to 46 of the most common conditions that affect humans [44]. There is a significantly high chance of any individual acquiring at least one of these diseases in their lifetime, so a wearable with a combination of sensors has a high value. Further, on top of clinical diagnostics, these devices can be used for other purposes. For example, an EMG allows for the control of prosthetics and can even act as a control to interact with Application and Human Computer Interfaces. A prime example of this is the MYO controller that is currently in the market and being used in lifestyle applications and as an HCI (Human computer Interaction) device. Moreover, the MYO controller has been proven to provide clinically comparable data [5]. Another major scenario for EMG that requires the use of the IoT is motor rehabilitation programs. The combination of these devices in an IoT framework will cover at least 70% of the disease diagnosis without the need for the physical presence of the patient with the doctor.

As stated above, we have managed to investigate possible hardware designs for biomedical wearables, the components they require, and some of their limitations. The future of wearables seems promising and there is significant potential in designing and developing wearables for various applications. At the speed the industry is growing, there will only be further improvements in the security and technological aspects. One of the major contributing factors to that is the collective interest of governments, industry, and the academic community. As a matter of fact, the results and designs outlined in this paper will facilitate and speed up the research being done on machine learning and Artificial Intelligence (AI) systems for informed decision-making in healthcare technology and delivery [45].

## 5. Textile Wearables

### 5.1. Electronic Textiles

Ever since humans have become civilized, we have used natural materials, such as cotton and silk, in the form of woven textiles to stay warm and comfortable. As demand has increased for certain qualities in textiles, manmade materials, such as Kevlar and nylon, have appeared in the market over the last century [28]. Since the advancement of technology and primarily the internet, as mentioned in the Introduction, textiles have also faced new challenges. They are now expected to possess additional features and functionalities besides those already bestowed on them [46]. Features, such as charging your phone by storing electric energy in clothes, and, hence, different functionalities have been incorporated into these materials. Some common examples are photoactive materials being integrated into textiles to create solar cells that create electricity and sensing materials being integrated into fibers to detect shape changes [46]. Electronic textiles (E-textiles, smart textiles, functional fabrics) are fabrics that allow for digital components and electronics to be embedded in them [47]. They are soft and flexible, allowing them to be in close contact with the curved surfaces of the body [46]. This is a fundamental property for all wearable devices. Contact with the body is necessary for signal acquisition, and e-textiles increase the surface that is in contact with the body. Furthermore, electronic textiles are lighter and more breathable when compared to their traditional sensor counterparts. As a matter of fact, the human factors with fabrics are also increased significantly. Fabrics are soft, while traditional biomedical wearables are solid and bulky even if they are reduced down to a flexible printed circuit board (PCB). Further, if they are reduced down to one plane, they can deform by bending or twisting motions. Probably one of the most important advantages that fabric wearables have is the human factors that go along with them. Traditional wearables are “add-ons” or accessories that must be worn on the person as an additional step after clothes are worn. This decreases the chances of a successful wearable, as human factors are reduced and the adoption rate is also reduced, as was mentioned in the Factors to Consider section. This is where electronic textiles or functional fabrics play an important role.

**Figure 13:**
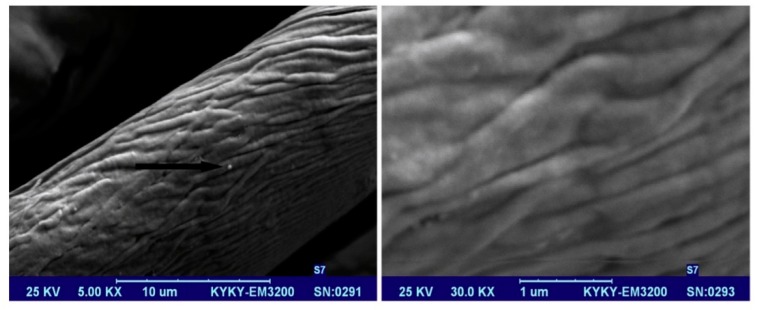
An electroconductive cotton fiber coated with graphene using the “dip-and-dry” method. Image source: [47].

As with the design of any wearable, these wearable textiles require conducting pathways for the signals. This is done using conducting and semi-conducting materials. There are several ways to produce these fabrics, such as using metallic fibers or strands that are mixed in with the fabric to create electroconductive textiles or semi-conductive textiles. They utilize conducting polymers, such as carbon nanotubes, metal-based powders, and carbon black [47]. The fabrics that are produced by these methods have some disadvantages, such as requiring complex processes, lacking a uniform coating, not having durable wear resistance, and not being entirely flexible [47]. This led to the use of graphene in these fabrics. Graphene is a honeycomb lattice of carbon atoms that exhibits outstanding electronic, thermal, mechanical, and optical properties [47]. For this reason, it has attracted significant attention for use in e-textiles. Furthermore, large quantities of graphene can be prepared from the chemical conversion of graphite using reduction methods. For example, to create electroconductive materials, graphene oxide is deposited on the surface of fabrics using the ‘dip-and-dry’ method. However, this leads to an electrically insulating material, and reduction methods are employed to restore its electrical conductivity [34]. Figure 13 below demonstrates the dip-and-dry method.

### 5.2. Current Electronic Textiles in the Market

**Figure 14:**
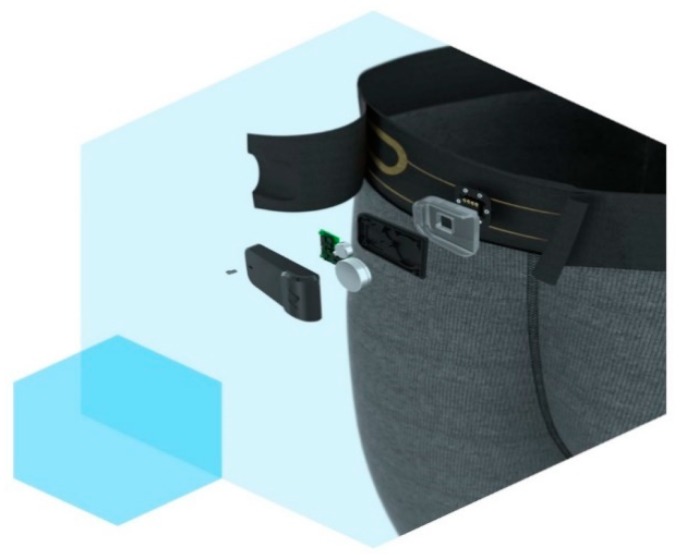
A current Skiin product by Myant. Image source: [48].

With these developments in textiles, several companies have started commercializing these e-textiles. One such example is Myant, a Canadian company that is a leader in functional fibers. They currently have a brand by the name of Skiin that has high performance smart underwear. The underwear is capable of measuring heart rate, breathing patterns, and temperature, and can track sleep, all day activity, and exercise [48]. It comes with a Skiin Companion App that connects to the device for bio-tracking. The current generation device costs 279 CAD, and all of the electronics are attached on the waist band in a removable compartment that is shown in Figure 14. That being said, they are releasing their next generation with functional fabrics. They have incorporated physiological sensors into their garments using advanced knitting techniques, and are now moving towards the introduction of Radio-Frequency Identification technology (RFID) [49]. It has been proven recently that the use of RFID technology for traditional metal-based tags allows for the capture of material deformations. The theory behind this is that, as the antenna inside the tag deforms as forces are applied, there is a change in the resonant frequency. This change is then received and correlated to a mechanical deformation on the object the tag is on [35]. This allows for a wearable with an incorporated strain sensor that is wireless and battery-less and, most importantly, comfortable to wear. This technology is not cheap, as it is currently in the developmental stages. The previous version that has physical sensors on the underwear costs around $280, and this new wearable could be more expensive. This technology does have vast amounts of potential; however, research and comparative studies will need to be performed to validate the signals that are retrieved from electronic textiles when compared to medical grade signals.

## Figures and Tables

**Table 1:** The frequency range of common biomedical signals.

Signal	Frequency Range (Hz)
PPG	0.5–5 ^1^
EMG	50–150 ^1^
Cardiac Auscultation	20–420 ^1^
Gait Analysis	0–15 ^1^

^1^ Ref. [5,8,12,13] respectively.

**Table 2:** The required Materials and Specifications.

Device	Operating Voltage	Inbuilt ADC	Supply Current	Output Voltage Range	Cost ^2^ (CAD)
Pulse Sensor (SEN-11574)	3–5.5 V	N/A ^1^	3–4 mA	0.3–5 V	24.95
EMG Sensor (SEN-0240	3.3–5.5 V	N/A	20 mA	0–3 V	50.03
Electret microphone (CEM-C9745JAD462P2.54R)	1–10 V	N/A	0.5 mA	≤10 V	0.95
MPU9250	2.4–3.6 V	16 bit	450 μA–3.2 mA	2.4–3.6 V	14.95
Arduino Uno	6–20 V	10 bit	20–50 mA	N/A	35.95
HC-05 Bluetooth Module	3.3–5 V	8 bit transfer	≈35 mA	N/A	11.99
Raspberry PI Zero W	5 V	11–17 bit	1.2 A	N/A	28.95
Batteries	N/A	N/A	400–600 mAH	9 V	1.47

References [6,7,18,33,34,35,36], ^1^ N/A: Not available, ^2^ Cost as of 30 September 2018.

**Table 3:** (**a**) Shows the connections for the first two sensors. (**b**) Shows the connections for the last two sensors.

**(a)**							
	**Pulse Sensor**				**EMG Sensor**		
Sensor	Black (ground)	Red (power)	Purple (Signal)		−(ground)	+(power)	A (Signal)
Arduino	GND	5 V	A0 (Analog in)		GND	5 V	A0 (Analog in)
**(b)**							
	Electret Microphone			IMU			
Sensor	Black (ground)	Red (power)	Red (power)	VDD	GND	SCL	SDA
Arduino	GND	5 V	A0 (Analog in)	5 V	GND	A5 (Analog in)	A4 (Analog in)

GND, ground; VDD, power supply; SDA, serial data; SCL, serial clock.
